# Characteristic Changes of Prefrontal and Motor Areas in Patients with Type 2 Diabetes and Major Depressive Disorder During a Motor Task of Tai Chi Chuan: A Functional Near‐Infrared Spectroscopy Study

**DOI:** 10.1002/brb3.70071

**Published:** 2024-10-08

**Authors:** Jiaming Zhang, Yuxi Li, Xiaobo Liu, Dongling Zhong, Chen Xue, Jin Fan, Cheng Xie, Juan Li, Rongjiang Jin

**Affiliations:** ^1^ School of Health Preservation and Rehabilitation Chengdu University of Traditional Chinese Medicine Chengdu China; ^2^ Department for Neural Function Detection and Regulation West China Xiamen Hospital, Sichuan University Xiamen China; ^3^ School of Acumox and Tuina Chengdu University of Traditional Chinese Medicine Chengdu China

**Keywords:** brain activation | functional connectivity | lateralization | major depressive disorder | tai chi chuan | type 2 diabetes mellitus

## Abstract

**Aim:**

This cross‐sectional study aims to identify the characteristic changes of prefrontal and motor areas during a tai chi chuan task in patients with Type 2 diabetes mellitus (T2DM) and major depressive disorder (MDD) using wearable functional near‐infrared spectroscopy (fNIRS).

**Methods:**

Three parallel groups (T2DM with DD group, T2DM group, and healthy group) were recruited from December 10, 2022, to May 31, 2023. Participants in three groups conducted a motor task of tai chi chuan designed by Eprime 3.0, and fNIRS was used to monitor the brain activation, functional connectivity (FC), and lateralization of prefrontal and motor areas. Correlation analyses were performed to examine the relationship between depressive symptoms and the function of prefrontal and motor areas.

**Results:**

Ninety elder adults (aged ≥ 60), including 30 patients with T2DM and MDD, 30 patients with T2DM, and 30 healthy subjects, were enrolled. In contrast with the patients with T2DM and healthy subjects, the patients with T2DM and MDD had decreased activation and abnormal lateralization in prefrontal and motor areas and decreased FC among supplementary motor area, motor area, and dorsolateral prefrontal cortex (DLPFC). Furthermore, the oxyhemoglobin (HbO_2_) concentration value of DLPFC in patients with T2DM and MDD was negatively associated with scores of Hamilton Depression Scale‐24 (HAMD‐24).

**Conclusions:**

Patients with T2DM and MDD had characteristic functional changes in prefrontal and motor areas. DLPFC may be a potential target of diagnosis and intervention for patients with T2DM and MDD.

## Graphical abstract

Schematic overview of the data analysis pipeline: (A) preparation before fNIRS task—including subject recruitment, scale evaluation, and learning tai chi chuan; (B) fNIRS task—subjects received fNIRS assessment when conducting tai chi chuan task; (C) data analysis—differences of activation, lateralization, and functional connectivity among three groups.

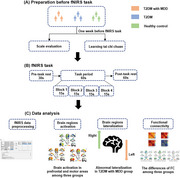

## 1 Introduction

Type 2 diabetes mellitus (T2DM) and major depressive disorder (MDD) are highly prevalent and frequently coexist in older adults (Semenkovich et al. 2015). Depressive mood is associated with reduced daily activities, increased social stigma, and lower self‐care ability in patients with T2DM (Tomic, Shaw, and Magliano 2022; Shoji et al. 2024; Dadras, Molaei, and Aghamohammadi 2022). The prevalence of MDD among individuals with T2DM has been reported to be as high as 28% (Harding et al. 2019). T2DM and MDD interact and create a vicious cycle, resulting in a significant health issue (Mukherjee and Chaturvedi 2019). Practice guidelines from the American Diabetes Association and the World Health Organization recommend that physicians should pay attention to depressive symptoms in patients with T2DM (Owens‐Gary et al. 2019). At present, subjective depression measurements, including structured clinical interview, self‐rating and other‐rating of depression scale, were usually used in clinical practice to assess the depressive status of T2DM patients (Mata et al. 2015).

The prefrontal cortex played a crucial role in emotion management, and the depressive mood was associated with reduced oxyhemoglobin (HbO_2_) of the prefrontal cortex in patients with MDD (Huang et al. 2022). Sun et al. (2017) monitored the activation of prefrontal cortex in patients with T2DM using functional magnetic resonance imaging (fMRI), and the results showed that, different from healthy subjects, patients with T2DM had decreased activation in the ventral medial prefrontal cortex (VMPFC) and orbitofrontal area (OA), whereas they manifested hyperactivity in the dorsolateral prefrontal cortex (DLPFC). Moreover, the net scores during the Iowa Gambling Task (IGT) were positively correlated with changes in brain activation of the VMPFC and OA. The net scores of IGT in T2DM patients were lower than healthy controls. Thus, Sun et al. speculated that T2DM patients might suffer from inhibition in their prefrontal cortex functions. Engels et al. (2010) found that depression was associated with rightward frontal lateralization in the DLPFC. However, the functional changes and lateralization of prefrontal cortex in patients with T2DM and MDD were not well investigated. In addition, Salustri et al. (2007) monitored the cortical excitability of the cerebral areas devoted to hand control in 12 patients with MDD, and the results demonstrated that primary sensory cortex (PSC) excitability appeared significantly reduced in MDD patients, and excitability was higher in the right hemisphere than that in the left. Therefore, we proposed a hypothesis that patients with T2DM and MDD had abnormal lateralization in both prefrontal and motor areas.

Tai chi chuan, as a multimodal mind–body exercise, is becoming increasingly popular worldwide. It incorporates physical, psychological, social, and meditative components, which promote the health of body and mind (Wang et al. 2021; Zhou et al. 2019). Previous studies demonstrated that participants showed characteristic brain changes after practicing tai chi chuan. Qi et al. (2023) found that the prefrontal and motor areas had characteristic changes in elder adults during tai chi standing meditation. Another study conducted by Qi et al. (2024) revealed that the hemodynamic activity of DLPFC and ventrolateral prefrontal cortex in older adults increased after 6 min of tai chi chuan training. Moreover, Kilpatrick et al. (2022) used resting‐state fMRI to observe the brain connectivity changes after 12 weeks’ tai chi chuan exercise; the results suggested that the functional connectivity (FC) of the default mode network enhanced in patients with geriatric depression. As a result, tai chi chuan can be used as a detection task to investigate the brain functional changes in patients with T2DM and MDD.

Functional near‐infrared spectroscopy (fNIRS) is based on the neurovascular coupling mechanism to obtain real‐time concentration variations of HbO_2_, deoxyhemoglobin (Hb), and total hemoglobin (THbO_2_) in the cerebral cortex (Pinti et al. 2020). As a noninvasive neuroimaging technology, fNIRS has moderate temporal–spatial resolution and can accurately reflect hemodynamic changes of cerebral hemispheric in participants (Chen et al. 2021). Furthermore, in comparison with other assessment techniques of brain function, wearable fNIRS is able to evaluate brain function alterations of participants during exercise, thus enabling researchers to comprehensively assess the differences of brain function in motor states (Schmaderer et al. 2023). Chen et al. (2022) found that tai chi chuan could increase the activation of the bilateral prefrontal cortex in elder adults with MDD based on fNIRS data. Wang et al. (2021) also revealed that, compared with non‐MDD subjects, patients with MDD have a weaker brain FC based on fNIRS data.

In this study, we planned to compare the differences of brain activation, lateralization, and FC in the prefrontal and motor areas among patients with T2DM and MDD, patients with T2DM, and healthy subjects during the tai chi chuan task. Moreover, the association between depressive symptoms and the function of prefrontal and motor areas in patients with T2DM and MDD was explored, aiming to identify the characteristic changes of prefrontal and motor areas in patients with T2DM and MDD during the tai chi chuan task.

## 2 Methods

### 2.1 Study Design and Setting

Three parallel groups (T2DM with MDD group, T2DM group, and healthy group) were designed in the cross‐sectional study. This cross‐sectional study was conducted at the Affiliated Sichuan Provincial Rehabilitation Hospital of Chengdu University of TCM between December 10, 2022, and May 31, 2023. All participants provided written informed consents. The study was reported following the Reporting of Observational Studies in Epidemiology (STROBE) reporting guideline. The completed STROBE checklist is shown in Section S1.

### 2.2 Participants

We recruited patients with T2DM from the outpatient department of the Affiliated Sichuan Provincial Rehabilitation Hospital of Chengdu University of TCM. All patients were assessed by two experienced physicians and met the diagnostic criteria of T2DM issued by the American Diabetes Association (2013). The inclusion criteria of the T2DM patients were as follows: (1) age ranged from 60 to 75 years; (2) Montreal Cognitive Assessment (MOCA) test > 26; (3) no engagement in regular exercise in the last 3 months; (4) right‐handed; and (5) Berg balance scale (BBS) > 40; (6) participants were naive to tai chi chuan prior to the study. Patients were divided into T2DM with MDD group and T2DM group based on the diagnostic criteria of MDD. The diagnosis of MDD was assessed by two psychiatrists according to the Diagnostic and Statistical Manual of Mental Disorders, 5th ed. (DSM‐5) (Beech, Miner, and Thornton 2016). The exclusion criteria for both patients with T2DM and patients with T2DM and MDD were (1) any psychotic disorders other than MDD; (2) significant medical or neurological illness; (3) unsuitable for tai chi chuan; (4) skin damage in the head and forehead; and (5) illiterate (unable to use words or phrases).

Healthy subjects were recruited from the nearby communities by poster advertisement. The inclusion criteria for healthy controls were (1) age ranged from 60 to 75 years; (2) MOCA test > 26; (3) no engagement in regular exercise in the last 3 months; (4) right‐handed; (5) BBS > 40. Exclusion criteria included the same medical and psychiatric factors used to recruit patients, as well as T2DM and any psychiatric disorders or known history of significant psychiatric illness among first‐degree relatives.

### 2.3 Procedures

One week before the fNIRS task, the certified trainers with at least 5 years of experience instructed participants to practice four movements of tai chi chuan: “left pucker tail,” “right pucker tail,” “single whiplash,” and “cloud hand.” After the instruction, the trainers evaluated the participants’ movements from five aspects: movement accuracy (20 points), center of gravity transfer (20 points), range of motion (20 points), movement proficiency (20 points), and movement coordination (20 points). Participants who got more than 80 points were considered to be qualified. Unqualified participants were instructed again and received reassessment of tai chi chuan, and if they failed again, they would be excluded.

**FIGURE 1:**
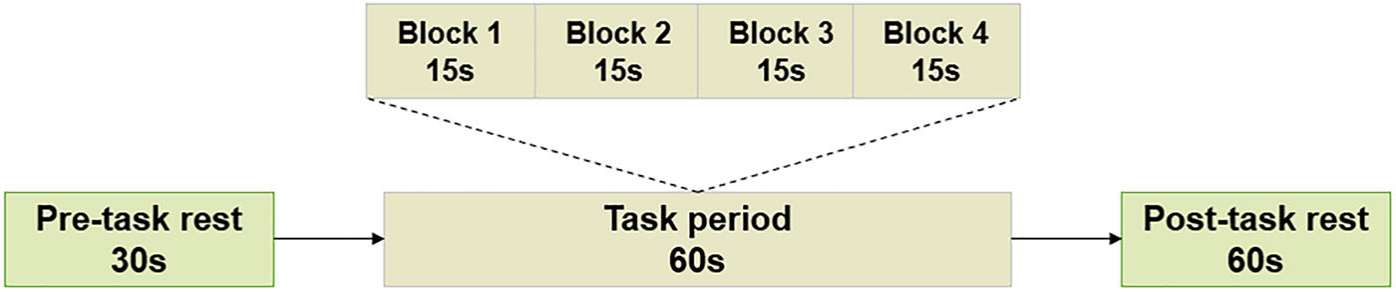
The fNIRS task paradigm. The tai chi chuan task protocol used for fNIRS. The task consisted of a 30‐s pre‐task rest period, a 60‐s task period subdivided into four 15‐s blocks and finally, a 60‐s post‐task rest period.

We used Eprime 3.0 (Psychology Software Tools Inc., Pittsburgh, USA) to design a fNIRS task (Figure 1). The task was divided into three periods: (1) Pre‐task rest period—participants were required to wear a detection cap and be seated peacefully, then they were required to follow the voice instructions from the fNIRS machine to verbally count the numbers for 30 s; (2) task period—the phase lasted for 60 s, including four sequential 15‐s blocks. During each 15‐s block, the subjects were asked to listen to the voice instructions from the fNIRS machine and complete the four movements of tai chi chuan; (3) post‐task rest period—participants followed voice instructions and verbally counted numbers for 60 s.

### 2.4 fNIRS Data Acquisition

**FIGURE 2:**
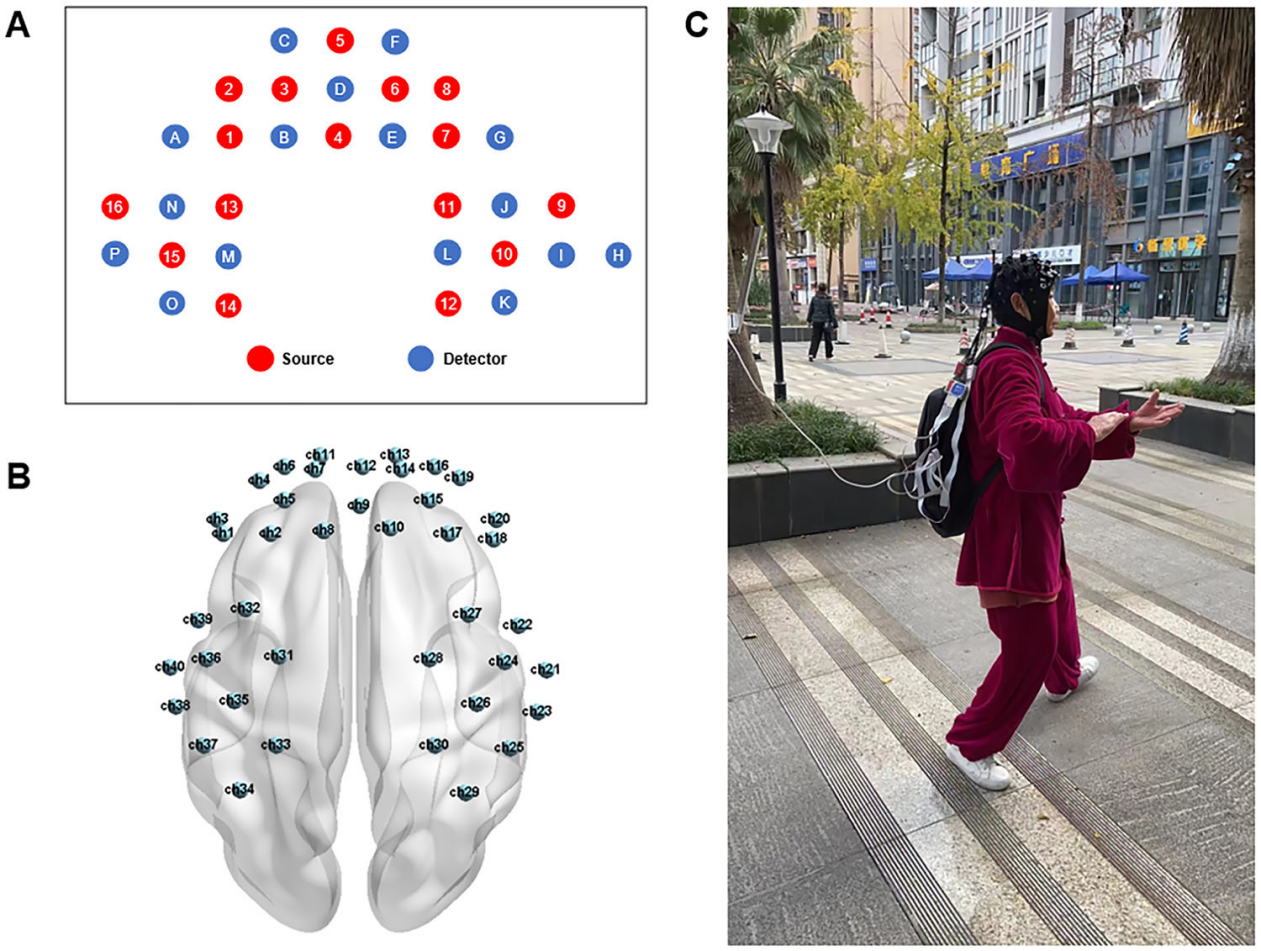
The device layout and test procedure of fNIRS: (A) light source and detector arrangement; (B) location of channels on standard head casts; (C) participant is being tested.

The fNIRS data were collected by the NIRSport devices (NIRx Medical Technologies LLC, Germany, USA). The fNIRS system used the 16 × 16 prefrontal cortex and motor area layout with 40 channels (CH). The source illumination type was light‐emitting diode. According to the international 10–20 system, each channel of the near‐infrared signals was collected from a source–detector (S–D) pair at the separation of 2.5 cm. A total of 20 channels were located in the left cerebral hemisphere (CH1–CH9, CH11, and CH31–CH40), and 20 channels covered the right cerebral hemisphere (CH10 and CH12–CH30). The sampling rate was 5 Hz. The fNIRS system monitored HbO_2_, Hb, and THbO_2_ of the prefrontal and motor areas using two wavelengths of near‐infrared light (760 and 850 nm). The fNIRS cap sizes were selected according to the head circumference of the participants and positioned in accordance with the automated anatomical labeling template. The cap provided an optimal contact between the surface of the probes and the scalp and a mini missing the likelihood of slips. Sources (*N* = 16) and detectors (*N* = 16) were manually placed after the cap was worn. Although there were slight variations in the S–D separation distances across caps, the distance was within the maximum optimal distance of 2.5 cm. Before the beginning of the acquisition, the collected fNIRS signals were adjusted by trained experimenters to assure that the validity was above 90%. In particular, the quality of the fNIRS signals was checked with the visual inspection tool provided by the NIRStar software (v15.2, Windows 64bit, a MATLAB‐based software provided by NIRx Medical Technologies LLC to acquire and record). The experimenters would adjust the coupling between the S–D separation, and the scalp before acquisition of data if the fNIRS channels had poor data quality according to the visual inspection tool. Furthermore, during the fNIRS task, an automatic calibration of the internal signal acquisition parameters (amplification gain) for each participant and channel was performed by the instrument. The validity of the collected signals for each period was assessed. Moreover, the standard Montreal Neurological Institute (MNI) coordinates and the Brodmann Area MRIcro area of 40 channels are shown in Section S2 (Table S1). The device layout and test procedure of fNIRS are presented in Figure 2.

### 2.5 Data Analysis

#### 2.5.1 Statistical Analysis of Clinical Outcome Measures

We used IBM SPSS 25.0 to analyze demographic data and the results of scale evaluation. The baseline indexes and scales assessment among the three groups were calculated by one‐way analysis of variance (ANOVA) or chi‐square test. The significance level was set as *p* < 0.05.

#### 2.5.2 fNIRS Data Preprocessing

The fNIRS data were preprocessed using the Homer2_UI software (Nguyen, Hoehl, and Vrtička 2021). The raw density of the data was converted into a densitometric value using the modified Beer–Lambert's law (MBLL). The differential pathlength factors (DPFs) were set to 5 and 6, respectively. The effect of errors in the DPFs was estimated by inverting the MBLL with wrong DPF values, which provided a maximum percentage error in the DPFs of 20%. Then, cubic spline corrections were performed to amend motion artifacts; the data were filtered using a band‐pass filter, and frequency bands from 0.01 to 0.1 Hz were selected for data analysis. Meanwhile, the value of optical density was converted into the value of hemoglobin concentration (including THbO_2_, HbO_2_, and Hb concentration), and the value of HbO_2_ concentration was extracted for analysis. The regions of interest (ROIs) in this study were the prefrontal cortex (DLPFC, Broca's area, frontopolar area [FPA], and OA) and motor area (supplementary motor area [SMA], primary motor cortex [PMC], PSC, frontal eye fields [FEF], superior temporal gyrus [STG], and supramarginal gyrus part of Wernicke's area).

#### 2.5.3 Activation Analysis of Brain Regions

The parameter estimates of the HbO_2_ concentration (*β* value) during the task execution phase (60 s in total) reflected the changes of activation in the prefrontal and motor areas. We used the NIRS_SPM toolkit in MATLAB 2013b to calculate parameter estimates (*β* values) of HbO_2_ concentration from 40 channels during the task period. The extent of brain activation and *β* value are positively correlated. A higher *β* value of a certain channel suggests a higher activation degree of the brain region. The *β* values were calculated to obtain *t* values for 40 channels in each group with a one‐sample *t*‐test (test value set to 0). The average *t* values for the corresponding channels were computed for ROI analysis. From CH1 to CH40, a one‐way ANOVA analysis on the *β* values among three groups within each channel was conducted, and post hoc analysis was performed between two groups. False discovery rate (FDR) was used for multi‐testing correction among 3 groups and 40 channels, respectively.

#### 2.5.4 Lateralization Analysis of Prefrontal and Motor Areas

To evaluate the asymmetry of the mean HbO_2_ in the bilateral prefrontal and motor areas during the task period (60 s), we calculated the laterality index (LI) as follows (Murayama, Hu, and Sakatani 2016):

$$\mathrm{LI}=\left(L-\mathrm{Hb}O_{2}-R-\mathrm{Hb}O_{2}\right)/\left(L-\mathrm{Hb}O_{2}+R-\mathrm{Hb}O_{2}\right)$$

The L‐HbO_2_ and R‐HbO_2_ denoted mean HbO_2_ concentrations of the left and right prefrontal and motor areas during the task period (60 s), respectively. A positive LI indicated a greater activation in the left prefrontal and motor areas, whereas a negative LI suggested a greater activation in the right prefrontal and motor areas. One‐way ANOVA was used to investigate the differences of LI in prefrontal and motor areas among three groups, and post hoc analysis with FDR was performed between two groups. Furthermore, LI ≥ 0.1 was defined as left‐sided lateralization, LI ≤ −0.1 as right‐sided lateralization, and LI between − 0.1 and 0.1 as symmetrical lateralization (Vernooij et al. 2007).

#### 2.5.5 FC Analysis

The FC existing in different brain regions could be reflected from both the time and frequency dimensions by a phase‐based FC (phase‐locking value, PLV) (Borgheai et al. 2020). In this study, PLV was adopted for FC intensity analysis, which quantified the level of synchronization among different signal phases. The PLV values among channels of each group during the period of task (60 s) were calculated, the difference of PLV values among three groups was detected using one‐way ANOVA, and then post hoc analysis was performed between two groups. The FDR was used for multi‐testing correction among 3 groups and 780 channel pairs, respectively.

#### 2.5.6 Correlation Analyses

To examine the relationship between the function of prefrontal and motor areas and depressive symptoms, correlation analyses between the HbO_2_ concentration, LI values, and PLV values of 40 channels and total scores of HAMD‐24 were performed, separately. If the data are normally distributed, Pearson correlation analyses are adopted; otherwise, Spearman correlation analyses are employed.

## 3 Results

### 3.1 The Demographic and Clinical Assessment

We recruited 30 patients with T2DM and MDD, 30 patients with T2DM, and 30 healthy subjects. The demographic information and clinical assessment of participants are summarized in Table 1. There were no statistical differences in baseline indexes, scores of Tai Chi movement (all > 80 points), and MOCA scores among three groups (*p* > 0.05). The patients with T2DM and MDD had higher scores of HAMD‐24 and SDSS than patients with T2DM and healthy subjects (*p* < 0.05). In addition, there was no difference between patients with T2DM and healthy subjects in scores of HAMD‐24 and SDSS (*p* > 0.05).

**TABLE 1:** The demographic characteristics, medical history characteristics, and the results of the scales assessment among three groups.

	T2DM with MDD group	T2DM group	Healthy group	Statistical value	*p* value
Age (years)	68.85 ± 3.67	67.90 ± 7.74	65.85 ± 3.18	*F* = 1.69	0.19
Sex (male/female)	12/18	15/15	13/17	*χ* ^2^ = 0.96	0.62
Height (CM)	163.1 ± 7.06	164.65 ± 6.89	161.45 ± 5.54	*F* = 1.20	0.31
Weight (KG)	55.85 ± 10.28	60.60 ± 7.37	58.40 ± 6.85	*F* = 1.64	0.20
Duration of T2DM (years)	10.08 ± 4.77	12.20 ± 3.98	—	*t* = − 1.53	0.13
Medication use in T2DM (none/1 specie/2 species/3 species and above)	5/22/3/0	0/30/0/0	—	*χ* ^2^ = 3.24	0.19
Alcohol consumption (yes/no)	2/18	7/13	5/15	*χ* ^2^ = 3.54	0.17
Smoking (yes/no)	7/23	11/19	10/20	*χ* ^2^ = 2.55	0.28
HAMD‐24	22.90 ± 2.67	3.25 ± 2.43^a^	3.75 ± 1.45^a^	*F* = 498.04	< 0.01
SDSS	4.45 ± 2.50	1 ± 1.45^a^	0.35 ± 0.59^a^	*F* = 33.45	< 0.01
MOCA	27.75 ± 0.55	27.60 ± 0.68	27.70 ± 0.66	*F* = 0.29	0.75
Tai chi chuan movement score	83.40 ± 1.05	83.30 ± 1.08	83.20 ± 1.11	*F* = 0.17	0.84

*Note*: a: The difference was statistically significant in comparison to T2DM with MDD group.

Abbreviations: MDD, major depressive disorder; MOCA, Montreal Cognitive Assessment; SDSS, Social Disability Screening Schedule; T2DM, Type 2 diabetes mellitus.

### 3.2 Prefrontal and Motor Areas Activation Analysis Among the Three Groups

#### 3.2.1 Overall Activation Analysis

As documented in Section S2 (Table S2), one channel (DLPFC) of patients with T2DM and MDD was activated during the tai chi chuan task (*p* < 0.05).

A total of four channels were activated (*p* < 0.05) in patients with T2DM; the activated brain areas were the prefrontal cortex (DLPFC, FPA, and OA) and motor area (PSC and Wernicke's area). Meanwhile, healthy subjects had nine activated channels (*p* < 0.05). The activated brain areas included prefrontal cortex (DLPFC, FPA, FEF, OA, and Broca's area) and motor area (PMC, STG, and SMA).

#### 3.2.2 Difference in Activation of Each Channel

The differences of mean *β* value in the channels among the three groups are presented in Section S2 (Table S3). The results showed that *β* values of 11 channels among 3 groups were statistically different (*p* < 0.05), and the corresponding brain areas involved FPA, OA, DLPFC, Broca's area, PMC, PSC, FEF, STG, and SMA.

**FIGURE 3:**
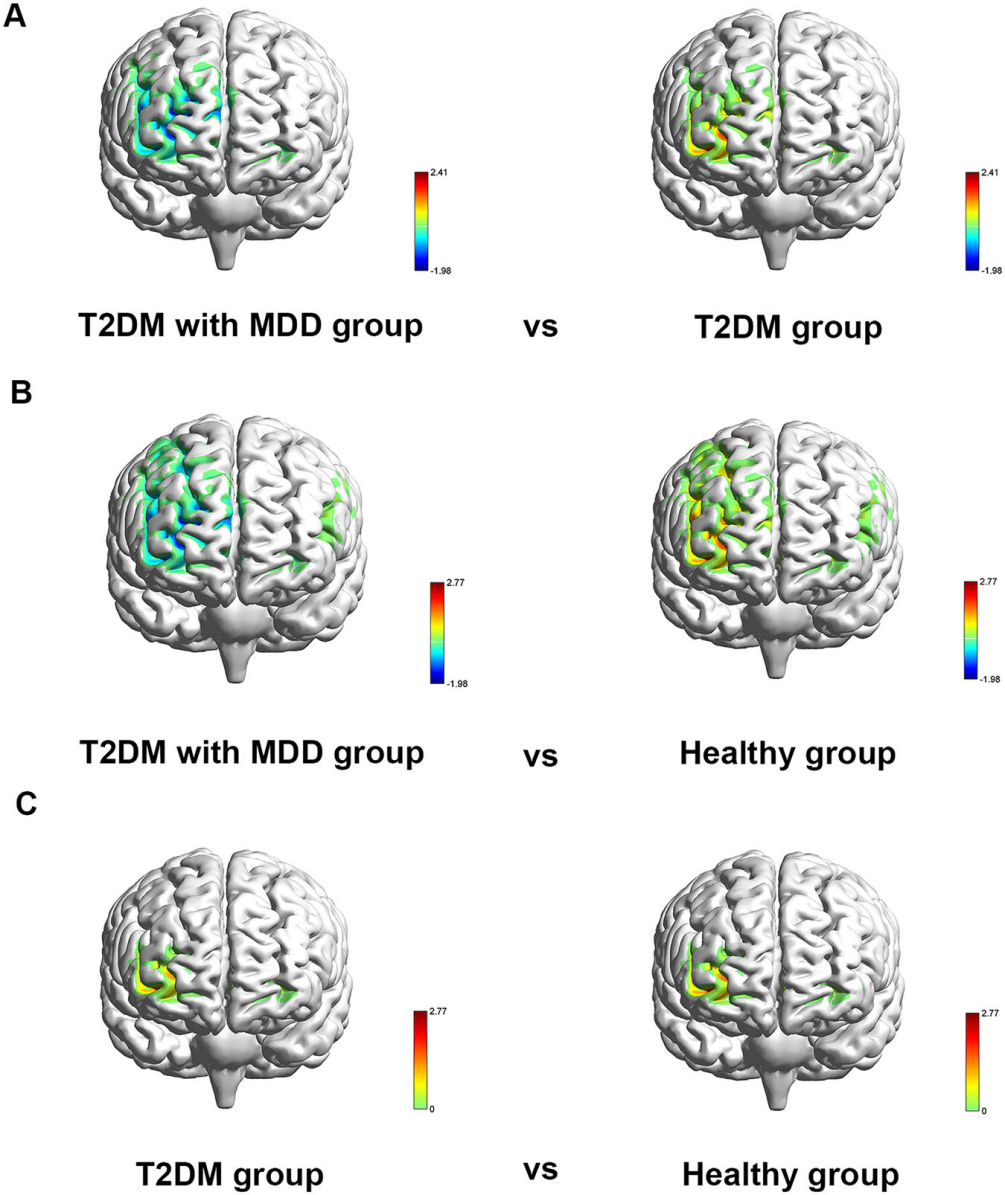
Activation in brain regions with significant differences among three groups (A–C). Warm shades represent positive activation, and cold shades represent negative activation. MDD, major depressive disorder; T2DM, type 2 diabetes mellitus.

The results of post hoc analysis indicated that, different from patients with T2DM, the averaged *β* values of three channels in patients with T2DM and MDD decreased (*p *< 0.01), and the negative activated brain areas were FPA, DLPFC, and Broca's area. Meanwhile, the averaged *β* values of five channels in patients with T2DM and MDD were lower than the healthy controls (*p* < 0.01); the corresponding brain areas included FPA, DLPFC, Broca's area, PMC, STG, and SMA. The averaged *β* values of one channel increased in healthy controls in comparison to patients with T2DM (*p* < 0.01), and the increased activation of brain regions was DLPFC. The aforementioned results are displayed in Figure 3.

### 3.3 Differences of Lateralization During the Tai Chi Chuan Task Among the Three Groups

#### 3.3.1 Differences of Lateralization Ratio Among the Three Groups

As shown in Section S2 (Table S4), the results indicated that the left‐sided lateralization ratio of the prefrontal and motor areas in patients with T2DM and MDD (prefrontal cortex: 37%, motor area: 13%) were lower than that in patients with T2DM (prefrontal cortex: 40%, motor area: 27%) and healthy controls (prefrontal cortex: 47%, motor area: 57%). In the meantime, patients with T2DM and MDD (prefrontal cortex: 60%, motor area: 60%) had a higher right‐sided lateralization ratio in the prefrontal and motor areas than patients with T2DM (prefrontal cortex: 47%, motor area: 57%) and healthy subjects (prefrontal cortex: 33%, motor area: 27%).

#### 3.3.2 Differences of Overall LI Among the Three Groups

The overall LI differences of prefrontal and motor areas are displayed in Section S2 (Table S5). The results showed that the overall LI in prefrontal and motor areas among three groups were statistically different (*p* < 0.05). The results of post‐hoc analysis manifested that, in contrast with healthy subjects, the overall LI of prefrontal and motor areas in patients with T2DM and MDD reduced (*p* < 0.05). Moreover, healthy subjects had higher overall LI in the motor area than patients with T2DM (*p* < 0.05).

### 3.4 Differences Analyses of FC

#### 3.4.1 Differences of FC Among Three Groups

**FIGURE 4:**
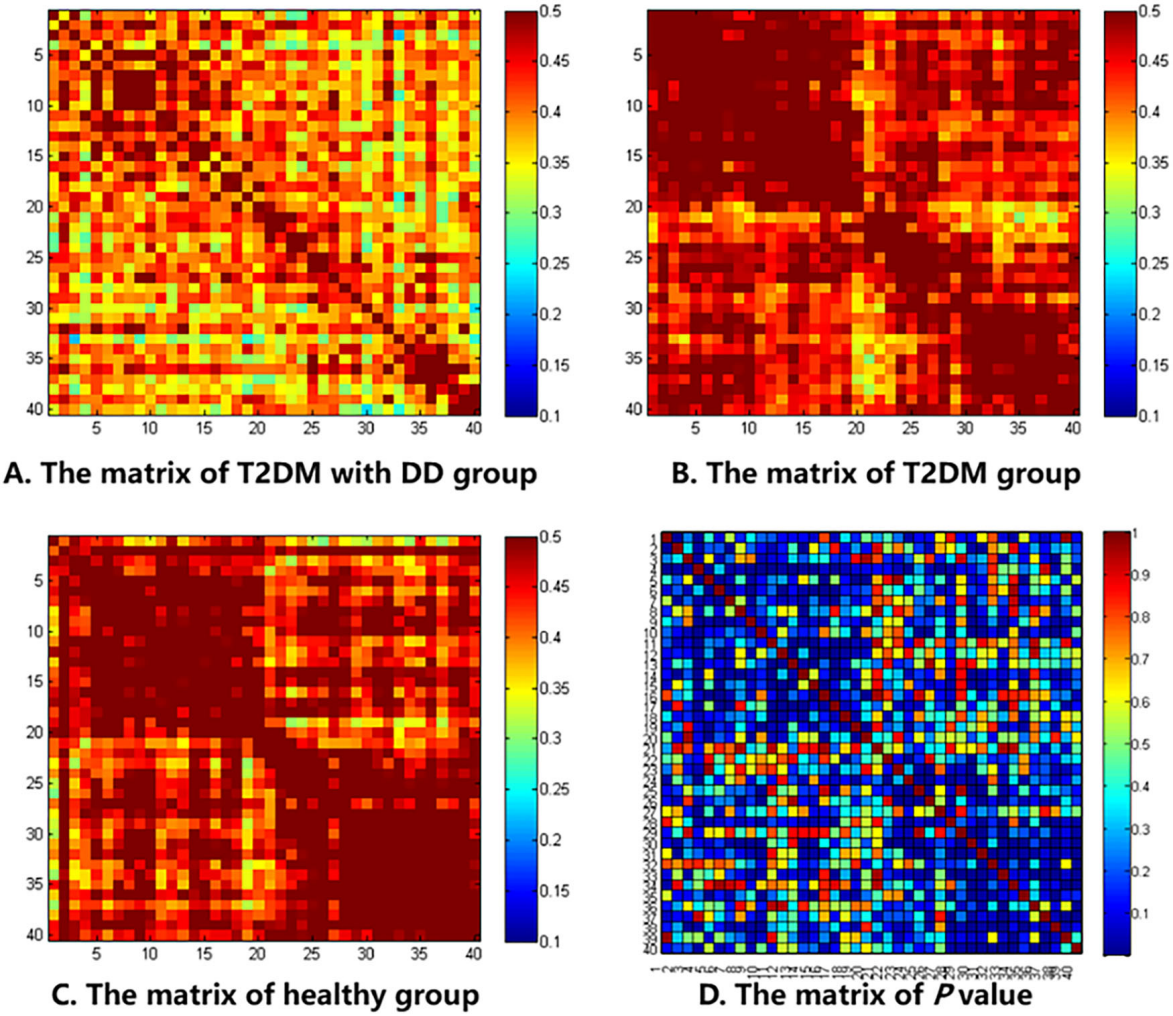
The PLV matrix among three groups and the *p* value matrix. (A) The PLV matrix of T2DM with MDD group. (B) The PLV matrix of T2DM group. (C) The PLV matrix of healthy group. (D) The PLV matrix of *p* value among three groups. The *x*‐ and *y*‐axis values in matrix figures were one‐to‐one correspondences to 40 channels, cells in the PLV matrix figures represent PLV value, and cells in the *p* value matrix figure represent statistical test values. MDD, major depressive disorder; T2DM, type 2 diabetes mellitus; PLV, phase‐locking value.

According to Section S2 (Table S6), the results demonstrated that there were 177 different pairs of channel connections among three groups (*p* < 0.05), the corresponding brain FC included within prefrontal cortex, DLPFC and SMA, DLPFC and motor area, within SMA, SMA and motor area, within motor area. The PLV matrix plots of the three groups and the *p* value matrix plot are shown in Figure 4.

#### 3.4.2 T2DM With MDD Group vs. T2DM Group

**FIGURE 5:**
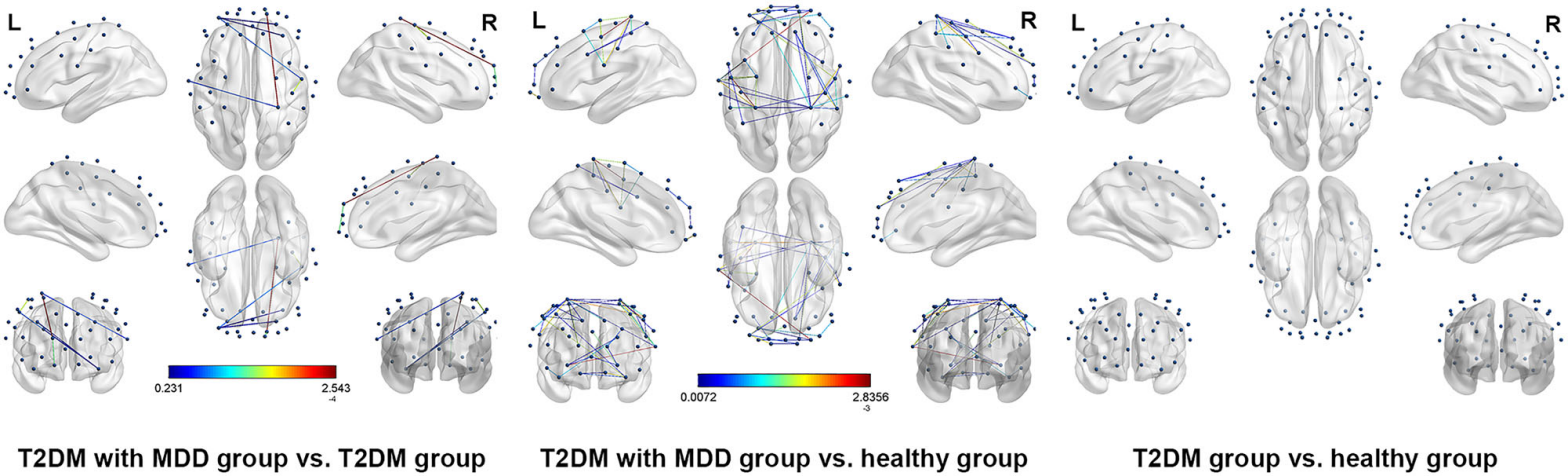
The FC differences among three groups. Nodes in the FC differences figure represent the layout of channels, whereas lines represent statistically significant FC from two groups. FC, functional connectivity; MDD, major depressive disorder; T2DM, type 2 diabetes mellitus.

As shown in Section S2 (Table S7), the results of post hoc analysis indicated that, different from patients with T2DM, seven pairs of channel connections decreased in patients with T2DM and MDD (*p* < 0.01), the corresponding brain FC involved within prefrontal cortex, SMA, and motor area. The results of FC in comparison of the patients with T2DM and patients with T2DM and MDD groups are presented in Figure 5.

#### 3.4.3 T2DM With MDD Group vs. healthy Group

Compared with the healthy controls, 45 pairs of channel connections in patients with T2DM and MDD decreased (*p* < 0.01), and brain regions with decreased FC included within the prefrontal cortex, DLPFC and SMA, DLPFC and motor area, SMA and motor area, and within the motor area.

#### 3.4.4 T2DM Group vs. Healthy Group

There was no difference in brain FC between T2DM patients and healthy subjects (*p* > 0.05).

### 3.5 Correlation Analyses

With regards to patients with T2DM and MDD, the results of Pearson correlation analysis showed that the HbO_2_ concentration value was negatively related to scores of HAMD‐24 in one channel (CH27), and the corresponding brain area was DLPFC (*r* = −0.45; *p* = 0.01). After excluding three outliers, the results indicated that there was no correlation between scores of HAMD‐24 and CH27 (*r* = −0.02, *p* = 0.92). The results of correlation analysis are shown in Section S2 (Figures S1 and S2). The HAMD‐24 scores were not correlated with LI values or PLV values of 40 channels (all *p* > 0.05).

## 4 Discussion

This is the first real‐time wearable fNIRS study to investigate the functional differences in prefrontal and motor areas of patients with T2DM with MDD in contrast with T2DM patients and healthy subjects during tai chi chuan tasks. The results showed that, different from patients with T2DM and healthy controls, patients with T2DM and MDD had lower activation of the prefrontal (FPA, DLPFC, Broca's area) and motor cortex (PMC, STG, SMA). The left‐sided lateralization ratio and LI of patients with T2DM and MDD decreased when compared to healthy controls. The FC among the prefrontal cortex, SMA, and motor cortex reduced in patients with T2DM and MDD when compared with patients with T2DM and healthy controls. Furthermore, a review (Kupferberg, Bicks, and Hasler 2016) concluded that the social disturbance of patients with MDD was pervasive and encompassed almost every aspect of one's social capabilities. Luo et al. (2024) found that even in clinical remission, part of patients with MDD still suffered from social dysfunction. Similarly, our findings showed that patients with T2DM and MDD had higher SDSS scores than patients with T2DM patients and healthy participants, which indicated that patients with T2DM and MDD had social dysfunction.

### 4.1 The Excitability of Prefrontal and Motor Areas Reduced in Patients With T2DM and MDD

Previous studies demonstrated that brain excitability of the prefrontal and motor areas in patients with MDD reduced in contrast with healthy subjects, which was related to symptoms like interest drops and decreased activity (Lefaucheur et al. 2008; Chao et al. 2021; Levinson et al. 2010). Similarly, in the present study, patients with T2DM and MDD exhibited lower activation in FPA than patients with T2DM and healthy individuals. Usami et al. (2014) revealed that enhanced activation in the FPA was associated with the improvement of depressive symptoms in adolescents with MDD. Furthermore, we found that patients with T2DM and MDD had a lower response in DLPFC than patients with T2DM and healthy subjects. Feng et al. (2021) revealed that compared with healthy individuals, patients with MDD had significant lower hemodynamic activation in the left DLPFC based on fNIRS data. Golkar et al. (2012) concluded that DLPFC could regulate emotion by reflection of the cognitive demand inherent to the emotional regulation task. Thus, the lower response of DLPFC in patients with T2DM and MDD was associated with the depressive mood.

Moreover, the activation of Broca's area in patients with T2DM and MDD was lower than in patients with T2DM and healthy subjects. Da et al. (2024) used fNIRS to monitor the activation of Broca's area during the verbal fluency task in patients with MDD. The results showed that there was a negative correlation between the activation in Broca's area and depressive symptoms. In addition, Cousineau et al. (2022) found that PMC was mainly responsible for movement execution. In the present study, the activation of PMC and STG reduced in patients with T2DM and MDD in contrast with T2DM patients and healthy participants. Thus, we speculated that patients with T2DM and MDD might be at a risk of dysfunction in perception and movement execution.

### 4.2 The Abnormal Lateralization of Prefrontal and Motor Areas in Patients With T2DM and MDD

Nusslock et al. (2018) suggested that MDD is related to reduced excitability in the left prefrontal cortex. An fMRI study also found that the FC of left ventral prefrontal cortex in patients with MDD decreased when compared with healthy subjects (Tang et al. 2013). In the present study, we found that the left‐sided lateralization ratio of prefrontal cortex in patients with T2DM and MDD was lower than patients with T2DM and healthy participants, whereas the right‐sided lateralization ratio showed a reverse result. Moreover, the overall LI of the prefrontal cortex in patients with T2DM and MDD was lower than in healthy subjects. This phenomenon suggested that patients with T2DM and MDD exhibited asymmetric function in the prefrontal cortex. Furthermore, Mottaghy et al. (2002) showed that prior to repetitive transcranial magnetic stimulation (rTMS) treatment, there was a significant left‐right asymmetry in the cerebral hemisphere favoring the right in patients with MDD, whereas such asymmetry was reversed after 2 weeks of rTMS treatment. Deslandes et al. (2010) revealed that pharmacological treatment plus aerobic training could reduce cortical activity on the right hemisphere and alleviate depressive mood in the elderly. The above results indicated that the depressive mood in patients with T2DM and MDD could be improved by correcting abnormal lateralization of the prefrontal cortex.

Additionally, right‐handers showed more asymmetrical activation in PMC, predominantly in the dominant (left) hemisphere (Bernard, Taylor, and Seidler 2011). However, we found that left‐sided lateralization ratio and overall LI of motor area in patients with T2DM and MDD were lower than healthy subjects. Cotovio et al. (2022) analyzed the excitability of motor areas in 60 patients with MDD and concluded that MDD patients had higher excitability in right motor areas compared with the left corresponding regions. Maeda, Keenan, and Pascual‐Leone (2000) used TMS to monitor the excitability of bilateral PMC in MDD patients, and the results showed that the patients had less excitability in the left motor cortex and had greater excitability in the right motor cortex than healthy controls. They speculated that patients with MDD had relatively low glutamatergic influence or excessive GABAergic tone in the left motor cortex by contrast with the right motor cortex.

In summary, our results indicated a rightward lateralization of prefrontal and motor areas in patients with T2DM and MDD, which were in line with previous studies on depression (Engels et al. 2010; Salustri et al. 2007). The above results effectively validated the previous hypothesis we proposed.

### 4.3 The FC Between Prefrontal and Motor Areas Decreased in Patients With T2DM and MDD

Our findings suggested decreased brain FC, including within the prefrontal cortex, DLPFC, and motor area, in patients with T2DM and MDD when compared with patients with T2DM and healthy controls. The DLPFC in both hemispheres had a central integrative function for motor control and behavior (Cieslik et al. 2013). Wang et al. (2020) showed that the left DLPFC inhibited the ipsilateral motor area and concluded that the inhibitory effect was related to indirect connections. On the basis of our findings, we speculated that the reduced FC of motor area in patients with T2DM and MDD might be the result of decreased FC in DLPFC. Moreover, SMA is reported to be associated with motor sequencing function and language function (Pinson et al. 2022). SMA integrates signals of frontal planning with sensorial, proprioceptive, and cognitive information from other brain regions and forwards it to the motor area to adjust muscles relevant to the intended movement (Marten et al. 2024). Our findings showed that the FC among SMA, prefrontal cortex, and motor area reduced in patients with T2DM and MDD, which indicated that impaired information was transferred from prefrontal cortex to motor area and was mediated by SMA.

To sum up, the present findings of altered activation, lateralization, and FC in prefrontal and motor areas complemented the previous studies to some extent and contributed to further understanding of the neuropsychological mechanism of MDD in T2DM patients.

### 4.4 Correlation Analysis

Usually, DLPFC was considered a key target to improve depressive symptoms in patients with MDD (Koutsomitros et al. 2021). A systematic review by Zhu et al. (2022) evaluated the effect of high‐frequency rTMS on the DLPFC for chronic pain complicated with depression, and the results showed that high‐frequency rTMS on the left DLPFC could relieve the depressive symptoms in patients with chronic pain. Furthermore, Blumberger et al. (2022) revealed that both standard bilateral rTMS and intermittent theta‐burst stimulation (iTBS) could improve depressive symptoms in elder patients with MDD. Our results revealed the correlation of the function in DLPFC with the depressive symptoms in patients with T2DM and MDD, which indicated that the DLPFC might be a potential target for diagnosis and intervention of MDD in T2DM patients. Intervention such as rTMS or iTBS on the DLPFC may be a promising therapeutic strategy for improvement of depressive symptoms in patients with T2DM and MDD. However, due to the unstable results of the correlation analysis, the above results should be treated with caution.

### 4.5 Limitations

We acknowledged several limitations in the present study. First, due to small vessel disease in T2DM and blood flow changes in MDD, the collected fNIRS signals in typical S–D channels might be possibly contaminated with systemic interference occurring in the superficial layers of the head. In order to remove systemic interference and improve the accuracy of fNIRS spectroscopy measurements, an additional short S–D separation optode should be used in future studies. Second, a single task block was used as an fNIRS task, and future studies should design a dual‐task paradigm to further detect changes of prefrontal and motor areas in patients with T2DM and MDD. Third, the patients with MDD were not recruited as a control group, so we were not able to investigate the activation in prefrontal and motor areas between patients with T2DM and MDD and patients with MDD. Fourth, the motor function–related scales were not assessed, and the motor function of the participants was unknown. Fifth, the examined brain regions in the present study involved the prefrontal and motor areas. The activation changes in other brain regions need further exploration.

## 5 Conclusion

We found that patients with T2DM and MDD showed reduced activation, abnormal lateralization index, and left‐sided lateralization ratio of prefrontal and motor areas, and decreased FC among prefrontal cortex, SMA, and motor area, which shed light on the neuropsychological mechanism of MDD in T2DM patients. Furthermore, DLPFC may be a potential target for diagnosis and intervention of MDD in T2DM patients. However, due to the limited sample size, the characteristic changes of prefrontal and motor areas in patients with T2DM and MDD are required for further exploration.

## Author Contributions

Jiaming Zhang, Yuxi Li, and Xiaobo Liu contributed to draft the manuscript. Rongjiang Jin and Juan Li participated in the design of the study. Dongling Zhong contributed to the interpretation of the data analyses. Chen Xue contributed to the recruitment of participants and collected the data. Jin Fan and Chen Xie participated in the design of the study. All authors have read and approved the final version of the manuscript and agreed with the order of presentation of the authors.

## Ethics Statement

The ethics committees of Affiliated Sichuan Provincial Rehabilitation Hospital of Chengdu University of TCM approved the study (grant no. CKLL‐2022032).

## Conflicts of Interest

The authors declare no conflicts of interest.

### Peer Review

The peer review history for this article is available at https://publons.com/publon/10.1002/brb3.70071.

## Supporting information



Supporting Information

Supporting Information

Supporting Information

Supporting Information

## Data Availability

The datasets generated during this study are available from the corresponding author upon reasonable request.
