# Supplementary material for: Characteristic Changes of Prefrontal and Motor Areas in Patients with Type 2 Diabetes and Major Depressive Disorder During a Motor Task of Tai Chi Chuan: A Functional Near‐Infrared Spectroscopy Study
Source: Brain Behav. 2024 Oct 8;14(10):e70071. doi: 10.1002/brb3.70071 (PMC11460607; doi:10.1002/brb3.70071)
Supplement: Supplementary file 2 — Supporting Information [file BRB3-14-e70071-s002.docx]

**Table S1 Positional information of each channel in the fNIRS device (mm)**

| Channel | | MNI coordinate mapping | | | Brodmann area | Percentage of overlap |
| --- | --- | --- | --- | --- | --- | --- |
|  | X | | Y | Z |  |  |
| CH1 | -47 | | 45 | 24 | 45-pars triangularis， Broca area | 0.76 |
| CH2 | -30 | | 45 | 43 | 44-pars opercularis, part of Broca's area | 0.88 |
| CH3 | -49 | | 50 | -1 | 46-DLPFC | 0.91 |
| CH4 | -34 | | 64 | -9 | 11-OA | 0.47 |
| CH5 | -25 | | 57 | 34 | 10, 11-FPA, OA | 0.51 |
| CH6 | -25 | | 69 | 4 | 11-OA | 0.63 |
| CH7 | -14 | | 68 | 24 | 10-FPA | 1 |
| CH8 | -11 | | 46 | 52 | 9-DLPFC | 0.78 |
| CH9 | 2 | | 55 | 40 | 9-DLPFC | 0.91 |
| CH10 | 13 | | 47 | 52 | 9-DLPFC | 0.78 |
| CH11 | -12 | | 73 | -4 | 11-OA | 0.56 |
| CH12 | 3 | | 69 | 13 | 10-FPA | 1 |
| CH13 | 15 | | 73 | -5 | 11-OA | 0.58 |
| CH14 | 17 | | 68 | 25 | 10-FPA | 1 |
| CH15 | 27 | | 57 | 35 | 9-DLPFC | 0.46 |
| CH16 | 26 | | 69 | 4 | 10-FPA | 0.66 |
| CH17 | 34 | | 45 | 43 | 9-DLPFC | 0.86 |
| CH18 | 50 | | 43 | 26 | 45-pars triangularis，Broca area | 0.89 |
| CH19 | 38 | | 65 | -9 | 9-DLPFC | 0.40 |
| CH20 | 51 | | 50 | 1 | 46-DLPFC | 0.85 |
| CH21 | 69 | | -4 | 25 | 3-FEF | 0.93 |
| CH22 | 59 | | 12 | 37 | 44-pars opercularis, part of Broca's area | 0.49 |
| CH23 | 66 | | -19 | 43 | 1, 4-PSC, PMC, SMA | 0.54 |
| CH24 | 54 | | -1 | 54 | 6-PMC | 0.97 |
| CH25 | 56 | | -32 | 57 | 1-PSC | 0.46 |
| CH26 | 44 | | -16 | 68 | 1-PSC | 0.59 |
| CH27 | 41 | | 16 | 59 | 9-DLPFC | 0.55 |
| CH28 | 27 | | 0 | 70 | 6-PMC | 1 |
| CH29 | 40 | | -48 | 68 | 7-Somatosensory Association Cortex | 0.41 |
| CH30 | 29 | | -31 | 75 | 4-PMC, SMA | 0.69 |
| CH31 | -27 | | 1 | 70 | 6-PMC | 1 |
| CH32 | -39 | | 18 | 59 | 9-DLPFC | 0.54 |
| CH33 | -28 | | -31 | 74 | 4-PMC, SMA | 0.73 |
| CH34 | -41 | | -47 | 67 | 40-Supramarginal gyrus part of Wernicke's area | 0.30 |
| CH35 | -43 | | -15 | 67 | 4-PMC, SMA | 0.61 |
| CH36 | -53 | | 0 | 53 | 6-PMC | 0.98 |
| CH37 | -54 | | -31 | 57 | 40-Supramarginal gyrus part of Wernicke's area | 0.41 |
| CH38 | -64 | | -17 | 41 | 1-PSC | 0.39 |
| CH39 | -56 | | 14 | 35 | 44- pars opercularis, part of Broca's area | 0.67 |
| CH40 | -66 | | -3 | 24 | 4, 8-PMC, SMA, STG | 0.88 |

Note: DLPFC: dorsolateral prefrontal cortex. FEF: frontal eye fields. FPA: frontopolar area. MNI: The standard Montreal Neurological Institute coordinates. OA: orbitofrontal area. PMC: primary motor cortex. PSC: primary somatosensory cortex. SMA: supplementary motor area. STG: superior temporal gyrus.

**Table S2 The activated channels in mean *β* values among 3 groups**

| Channel | Mean *β* value | Brodmann area | t value | | *P* value |
| --- | --- | --- | --- | --- | --- |
| **T2DM with MDD group** | | | | | |
| CH19 | 1×10^-5^±3×10^-5^ | 9-DLPFC | | 2.10 | 0.04 |
| **T2DM group** | | | | | |
| CH5 | 6.78×10^-8^±1.88×10^-7^ | 10, 11-FPA, OA | | 2.14 | 0.04 |
| CH19 | 9.15×10^-8^±9.16×10^-7^ | 9-DLPFC | | 2.77 | 0.01 |
| CH25 | 2.36×10^-7^±3×10^-5^ | 1-PSC | | 2.29 | 0.03 |
| CH37 | 1.20×10^-7^±6.48×10^-7^ | 40-Supramarginal gyrus part of Wernicke's area | | 2.33 | 0.03 |
| **Healthy group** | | | | | |
| CH2 | 1.24×10^-7^±3.21×10^-7^ | 44-pars opercularis, part of Broca's area | | 2.12 | 0.04 |
| CH5 | 8.48×10^-8^±2.06×10^-7^ | 10, 11-FPA, OA | | 2.26 | 0.03 |
| CH8 | 1.08×10^-7^±2.73×10^-7^ | 9-DLPFC | | 2.16 | 0.04 |
| CH11 | 2.43×10^-7^±5.19×10^-7^ | 11-OA | | 2.57 | 0.02 |
| CH12 | 1.34×10^-7^±3.44×10^-7^ | 10-FPA | | 2.14 | 0.04 |
| CH19 | 2.78×10^-7^±5.91×10^-7^ | 9-DLPFC | | 2.57 | 0.02 |
| CH20 | 2.44×10^-7^±5.55×10^-7^ | 46-DLPFC | | 2.41 | 0.02 |
| CH21 | 1.96×10^-7^±5.20×10^-7^ | 3-FEF | | 2.06 | 0.04 |
| CH40 | 2.03×10^-7^±5.36×10^-7^ | 4, 8-STG, PMC, SMA | | 2.08 | 0.04 |

Note: DLPFC: dorsolateral prefrontal cortex. FEF: frontal eye fields. FPA: frontopolar area. MDD: Major depressive disorder. OA: orbitofrontal area. PMC: primary motor cortex. PSC: primary somatosensory cortex. SMA: supplementary motor area. STG: superior temporal gyrus. T2DM: Type 2 diabetes mellitus.

**Table S3 The channels with differences in mean *β* value among the 3 groups**

| Channel | Mean *β* value | | | Brodmann area | F value | *P* value |
| --- | --- | --- | --- | --- | --- | --- |
|  | T2DM with MDD group | T2DM group | Healthy group |  |  |  |
| CH6 | 7.97×10^-8^±3.47×10^-7^ | 1.54×10^-7^±3.95×10^-5^ | 2×10^-5^±1×10^-4^ | 11-OA | 3.03 | 0.03 |
| CH14 | -1×10^-5^±3×10^-5^ | 4.23×10^-8^±2.49×10^-7 a^ | 5.19×10^-8^±3.21×10^-7 a^ | 10-FPA | 6.31 | <0.01 |
| CH17 | 6.03×10^-9^±3.81×10^-7^ | 3.73×10^-8^±5.26×10^-7^ | 7.05×10^-7^±2×10^-5^ | 9-DLPFC | 2.85 | 0.04 |
| CH18 | -2×10^-5^±1×10^-4^ | 2.46×10^-8^±4.38×10^-7 a^ | 1.19×10^-7^±3.38×10^-7 a^ | 45- Broca area | 4.43 | 0.01 |
| CH20 | -2×10^-5^±1×10^-4^ | 2.44×10^-7^±5.55×10^-7 a^ | 3.43×10^-7^±6.76×10^-7 ab^ | 46-DLPFC | 5.08 | <0.01 |
| CH21 | -7.61×10^-7^±3×10^-5^ | -9.97×10^-8^±1×10^-5^ | 1.96×10^-7^±5.20×10^-7^ | 3-FEF | 3.08 | 0.03 |
| CH23 | -1.63×10^-7^±2×10^-5^ | 9.34×10^-8^±3.13×10^-7^ | 1×10^-5^±4×10^-5^ | 1, 4-PSC, PMC, SMA | 2.87 | 0.04 |
| CH24 | -2.39×10^-7^±8.13×10^-7^ | 7.42×10^-8^±3.30×10^-7^ | 1.17×10^-7^±4.61×10^-7^ | 6-PMC | 3.13 | 0.03 |
| CH26 | -2.93×10^-7^±1×10^-5^ | 6.86×10^-8^±6.27×10^-7^ | 1.64×10^-7^±3.92×10^-7^ | 1-PSC | 3.21 | 0.02 |
| CH27 | -6.79×10^-8^±3.67×10^-7^ | 1.63×10^-7^±4.39×10^-7^ | 1×10^-5^±4×10^-5 a^ | 9-DLPFC | 3.55 | 0.02 |
| CH40 | 1.20×10^-7^±4.89×10^-7^ | 2.03×10^-7^±5.36×10^-7^ | 3×10^-5^±1×10^-4 a^ | 4, 8-STG, PMC, SMA | 3.29 | 0.02 |

Note: a: The difference was statistically significant compared with T2DM with the MDD group. b: The difference was statistically significant compared with the T2DM group. DLPFC: dorsolateral prefrontal cortex. FEF: frontal eye fields. FPA: frontopolar area. MDD: Major depressive disorder. OA: orbitofrontal area. PMC: primary motor cortex. PSC: primary somatosensory cortex. SMA: supplementary motor area. STG: superior temporal gyrus. T2DM: Type 2 diabetes mellitus.

**Table S4 Lateralization ratio differences of prefrontal and motor areas among 3 groups**

| Group | Brain area | left-sided lateralization (%) | right-sided lateralization (%) | symmetrical lateralization (%) |
| --- | --- | --- | --- | --- |
| T2DM with MDD group | Prefrontal cortex | n = 11 (37%) | n = 18 (60%) | n = 1 (3%) |
|  | Motor area | n = 4 (13%) | n = 18 (60%) | n = 8 (27%) |
| T2DM group | Prefrontal cortex | n = 12 (40%) | n = 14 (47%) | n = 4 (13%) |
|  | Motor area | n = 8 (27%) | n = 17 (57%) | n = 5 (16%) |
| Healthy group | Prefrontal cortex | n = 14 (47%) | n = 10 (33%) | n = 6 (20%) |
|  | Motor area | n = 17 (57%) | n = 8 (27%) | n = 5 (16%) |

Note: MDD: Major depressive disorder. T2DM: Type 2 diabetes mellitus.

**Table S5 Overall LI differences of prefrontal and motor areas among 3 groups**

| Brain area | Overall LI | | | F value | *P* value |
| --- | --- | --- | --- | --- | --- |
|  | T2DM with MDD group | T2DM group | Healthy group |  |  |
| Prefrontal cortex | −0.49 ± 1.29 | −0.13 ± 0.83 | 0.21 ± 0.53^a^ | 4.16 | 0.02 |
| Motor area | −0.25 ± 0.94 | 0.17 ± 1.19 | 1.37 ± 2.39^ab^ | 6.38 | <0.01 |

Note: a: The difference was statistically significant compared with T2DM with the MDD group. b: The difference was statistically significant compared with the T2DM group. LI: laterality index. MDD: Major depressive disorder. T2DM: Type 2 diabetes mellitus.

**Table S6 Channel connections that were statistically different among 3 groups during the tai chi chuan task**

| Channel connection | *P* value | Channel connection | *P* value | Channel connection | *P* value |
| --- | --- | --- | --- | --- | --- |
| CH1-CH2 | <0.01 | CH9-CH20 | 0.02 | CH22-CH30 | 0.01 |
| CH1-CH4 | <0.01 | CH9-CH30 | <0.01 | CH23-CH25 | 0.02 |
| CH1-CH24 | 0.02 | CH9-CH31 | 0.03 | CH23-CH26 | 0.01 |
| CH1-CH34 | 0.03 | CH9-CH33 | 0.02 | CH23-CH30 | <0.01 |
| CH2-CH4 | 0.02 | CH9-CH38 | 0.02 | CH24-CH25 | <0.01 |
| CH2-CH7 | <0.01 | CH10-CH19 | 0.01 | CH24-CH26 | <0.01 |
| CH2-CH11 | 0.04 | CH10-CH25 | <0.01 | CH24-CH28 | <0.01 |
| CH2-CH14 | <0.01 | CH10-CH26 | <0.01 | CH24-CH30 | <0.01 |
| CH2-CH30 | 0.03 | CH10-CH28 | 0.01 | CH24-CH33 | 0.03 |
| CH2-CH31 | 0.01 | CH10-CH30 | <0.01 | CH24-CH35 | 0.02 |
| CH3-CH4 | 0.02 | CH10-CH31 | 0.01 | CH25-CH26 | 0.01 |
| CH3-CH10 | 0.03 | CH10-CH33 | 0.03 | CH25-CH30 | <0.01 |
| CH3-CH33 | 0.02 | CH10-CH35 | <0.01 | CH25-CH31 | <0.01 |
| CH3-CH38 | <0.01 | CH10-CH37 | 0.04 | CH25-CH33 | 0.02 |
| CH4-CH5 | <0.01 | CH10-CH38 | 0.01 | CH26-CH28 | 0.02 |
| CH4-CH7 | 0.03 | CH11-CH13 | <0.01 | CH26-CH31 | <0.01 |
| CH4-CH8 | 0.04 | CH11-CH14 | <0.01 | CH26-CH33 | <0.01 |
| CH4-CH9 | <0.01 | CH11-CH15 | <0.01 | CH26-CH35 | 0.03 |
| CH4-CH10 | <0.01 | CH11-CH17 | <0.01 | CH26-CH37 | 0.02 |
| CH4-CH11 | 0.01 | CH11-CH18 | <0.01 | CH26-CH38 | 0.02 |
| CH4-CH12 | 0.02 | CH11-CH20 | 0.04 | CH28-CH31 | <0.01 |
| CH4-CH13 | <0.01 | CH12-CH13 | <0.01 | CH28-CH33 | <0.01 |
| CH4-CH14 | <0.01 | CH12-CH15 | <0.01 | CH28-CH35 | <0.01 |
| CH4-CH15 | <0.01 | CH12-CH16 | <0.01 | CH28-CH37 | <0.01 |
| CH4-CH17 | <0.01 | CH12-CH17 | <0.01 | CH28-CH38 | <0.01 |
| CH4-CH18 | 0.04 | CH12-CH18 | 0.03 | CH28-CH40 | 0.03 |
| CH4-CH24 | <0.01 | CH12-CH19 | 0.04 | CH29-CH31 | <0.01 |
| CH4-CH26 | 0.04 | CH13-CH14 | 0.02 | CH29-CH33 | 0.01 |
| CH4-CH27 | <0.01 | CH13-CH15 | <0.01 | CH29-CH38 | 0.03 |
| CH4-CH28 | 0.03 | CH13-CH17 | <0.01 | CH29-CH40 | 0.02 |
| CH4-CH40 | 0.02 | CH14-CH17 | <0.01 | CH30-CH31 | 0.02 |
| CH5-CH6 | <0.01 | CH14-CH18 | 0.02 | CH30-CH33 | <0.01 |
| CH5-CH9 | 0.02 | CH14-CH28 | 0.02 | CH30-CH34 | <0.01 |
| CH5-CH16 | <0.01 | CH14-CH30 | <0.01 | CH30-CH35 | <0.01 |
| CH5-CH19 | <0.01 | CH14-CH31 | 0.02 | CH30-CH37 | 0.02 |
| CH5-CH20 | <0.01 | CH14-CH38 | 0.03 | CH30-CH38 | <0.01 |
| CH5-CH28 | 0.03 | CH14-CH39 | 0.04 | CH30-CH40 | <0.01 |
| CH5-CH35 | <0.01 | CH15-CH16 | <0.01 | CH31-CH32 | <0.01 |
| CH6-CH7 | <0.01 | CH15-CH19 | 0.03 | CH31-CH33 | <0.01 |
| CH6-CH9 | <0.01 | CH15-CH28 | 0.03 | CH31-CH34 | 0.02 |
| CH6-CH12 | <0.01 | CH15-CH30 | 0.02 | CH31-CH35 | 0.02 |
| CH6-CH14 | 0.03 | CH15-CH31 | 0.02 | CH31-CH36 | 0.02 |
| CH6-CH15 | <0.01 | CH15-CH33 | 0.02 | CH31-CH38 | 0.02 |
| CH6-CH16 | 0.01 | CH15-CH35 | <0.01 | CH31-CH40 | <0.01 |
| CH6-CH17 | <0.01 | CH16-CH17 | 0.02 | CH32-CH33 | 0.04 |
| CH6-CH27 | 0.04 | CH16-CH20 | <0.01 | CH32-CH35 | 0.02 |
| CH7-CH9 | 0.03 | CH16-CH40 | 0.04 | CH32-CH38 | <0.01 |
| CH7-CH12 | 0.04 | CH17-CH23 | 0.04 | CH32-CH40 | <0.01 |
| CH7-CH14 | 0.02 | CH17-CH28 | <0.01 | CH33-CH36 | <0.01 |
| CH7-CH17 | <0.01 | CH17-CH33 | 0.01 | CH33-CH37 | <0.01 |
| CH7-CH30 | 0.03 | CH17-CH39 | 0.03 | CH33-CH38 | <0.01 |
| CH7-CH38 | 0.02 | CH18-CH19 | 0.02 | CH33-CH40 | <0.01 |
| CH8-CH14 | <0.01 | CH18-CH22 | 0.03 | CH34-CH39 | <0.01 |
| CH8-CH26 | <0.01 | CH18-CH35 | 0.03 | CH34-CH40 | 0.01 |
| CH8-CH28 | 0.03 | CH19-CH20 | <0.01 | CH35-CH38 | <0.01 |
| CH8-CH30 | <0.01 | CH19-CH24 | 0.02 | CH36-CH40 | 0.01 |
| CH8-CH38 | 0.03 | CH19-CH35 | 0.03 | CH37-CH39 | <0.01 |
| CH9-CH12 | 0.03 | CH19-CH38 | 0.02 | CH37-CH40 | <0.01 |
| CH9-CH16 | 0.03 | CH22-CH24 | <0.01 | CH38-CH39 | 0.03 |

**Table S7 Channel connections that were statistically different of back testing among 3 groups during the tai chi chuan task**

| **T2DM group vs. T2DM with MDD group** | | | | | | | |
| --- | --- | --- | --- | --- | --- | --- | --- |
| Channel connection | The PLV value in T2DM group | The PLV value in T2DM with MDD group | *P* value | Channel connection | The PLV value in T2DM group | The PLV value in T2DM with MDD group | *P* value |
| CH4-CH15 | 0.61 | 0.30 | <0.01 | CH14-CH30 | 0.52 | 0.31 | <0.01 |
| CH4-CH17 | 0.56 | 0.30 | <0.01 | CH24-CH26 | 0.72 | 0.38 | <0.01 |
| CH4-CH24 | 0.54 | 0.31 | <0.01 | CH30-CH40 | 0.51 | 0.24 | <0.01 |
| CH13-CH14 | 0.65 | 0.44 | <0.01 |  |  |  |  |
| **Healthy group vs. T2DM with MDD group** | | | | | | | |
| Channel connection | The PLV value in healthy group | The PLV value in T2DM with MDD group | *P* value | Channel connection | The PLV value in healthy group | The PLV value in T2DM with MDD group | *P* value |
| CH2-CH7 | 0.63 | 0.41 | <0.01 | CH24-CH25 | 0.67 | 0.42 | <0.01 |
| CH4-CH11 | 0.70 | 0.51 | <0.01 | CH24-CH26 | 0.66 | 0.38 | <0.01 |
| CH4-CH13 | 0.65 | 0.42 | <0.01 | CH24-CH30 | 0.56 | 0.29 | <0.01 |
| CH4-CH24 | 0.47 | 0.31 | <0.01 | CH25-CH26 | 0.71 | 0.51 | <0.01 |
| CH4-CH27 | 0.48 | 0.29 | <0.01 | CH25-CH30 | 0.63 | 0.37 | <0.01 |
| CH5-CH16 | 0.59 | 0.37 | <0.01 | CH26-CH33 | 0.58 | 0.29 | <0.01 |
| CH5-CH19 | 0.54 | 0.35 | <0.01 | CH30-CH33 | 0.62 | 0.29 | <0.01 |
| CH6-CH7 | 0.69 | 0.41 | <0.01 | CH30-CH34 | 0.59 | 0.37 | <0.01 |
| CH6-CH9 | 0.58 | 0.36 | <0.01 | CH30-CH35 | 0.51 | 0.31 | <0.01 |
| CH6-CH12 | 0.74 | 0.50 | <0.01 | CH30-CH37 | 0.53 | 0.33 | <0.01 |
| CH8-CH30 | 0.54 | 0.34 | <0.01 | CH30-CH40 | 0.45 | 0.24 | <0.01 |
| CH10-CH25 | 0.59 | 0.37 | <0.01 | CH31-CH32 | 0.69 | 0.42 | <0.01 |
| CH10-CH26 | 0.63 | 0.42 | <0.01 | CH31-CH33 | 0.57 | 0.36 | <0.01 |
| CH10-CH30 | 0.55 | 0.30 | <0.01 | CH31-CH36 | 0.62 | 0.41 | <0.01 |
| CH11-CH13 | 0.71 | 0.43 | <0.01 | CH31-CH40 | 0.53 | 0.30 | <0.01 |
| CH12-CH16 | 0.66 | 0.39 | <0.01 | CH32-CH40 | 0.55 | 0.34 | <0.01 |
| CH14-CH28 | 0.52 | 0.34 | <0.01 | CH33-CH36 | 0.62 | 0.39 | <0.01 |
| CH14-CH30 | 0.53 | 0.31 | <0.01 | CH33-CH37 | 0.64 | 0.40 | <0.01 |
| CH16-CH40 | 0.45 | 0.30 | <0.01 | CH33-CH38 | 0.61 | 0.32 | <0.01 |
| CH19-CH20 | 0.58 | 0.38 | <0.01 | CH33-CH40 | 0.48 | 0.26 | <0.01 |
| CH22-CH24 | 0.61 | 0.39 | <0.01 | CH34-CH39 | 0.57 | 0.38 | <0.01 |
| CH22-CH30 | 0.50 | 0.35 | <0.01 | CH37-CH39 | 0.55 | 0.35 | <0.01 |
| CH23-CH30 | 0.55 | 0.30 | <0.01 |  |  |  |  |
| **T2DM group vs. T2DM with MDD group** | | | | | | | |
| None |  |  |  |  |  |  |  |

Note: MDD: Major depressive disorder. PLV: phase locking value. T2DM: Type 2 diabetes mellitus.


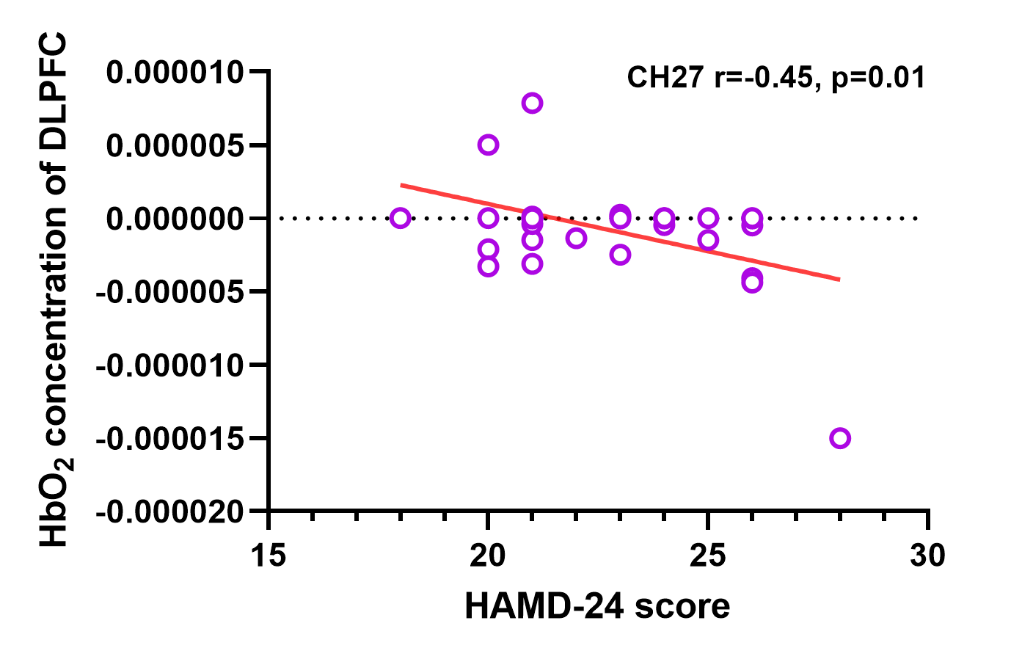


Fig. S1 The results of correlation between HbO_2_ concentration of DLPFC and HAMD-24 scores in patients with T2DM and MDD.

Note: CH: Channel. DLPFC: dorsolateral prefrontal cortex. HbO_2_: oxyhemoglobin. HAMD-24: Hamilton Depression Scale-24.


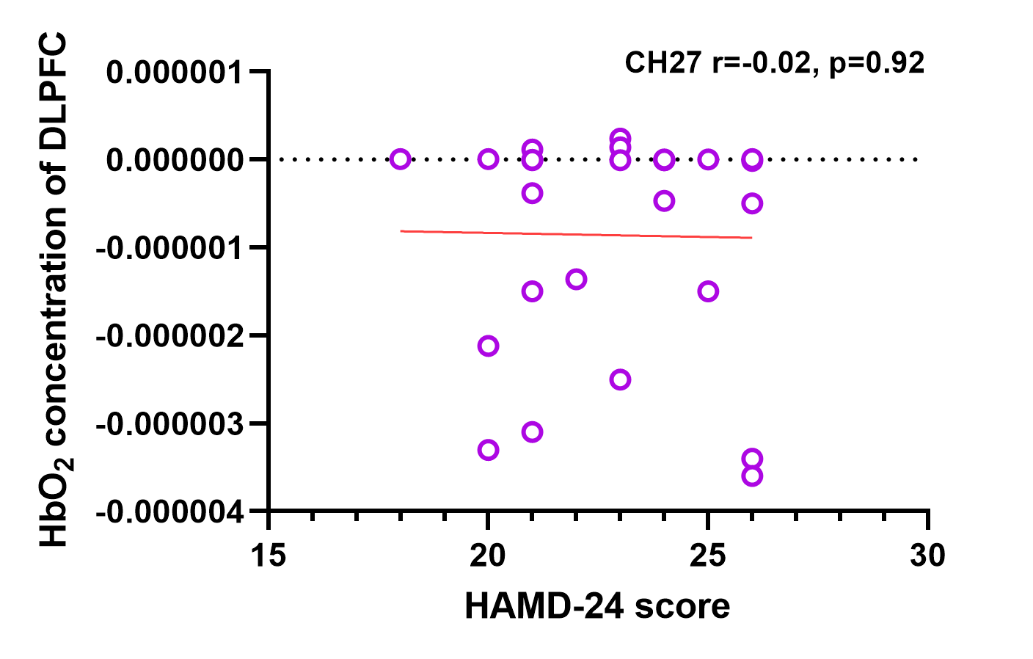


Fig. S2 The results of correlation between HbO_2_ concentration of DLPFC and HAMD-24 scores in patients with T2DM and MDD after excluding 3 outliers.

Note: CH: Channel. DLPFC: dorsolateral prefrontal cortex. HbO_2_: oxyhemoglobin. HAMD-24: Hamilton Depression Scale-24.
