# Supplementary figures and images for: Characteristic Changes of Prefrontal and Motor Areas in Patients with Type 2 Diabetes and Major Depressive Disorder During a Motor Task of Tai Chi Chuan: A Functional Near‐Infrared Spectroscopy Study
Source: Brain Behav. 2024 Oct 8;14(10):e70071. doi: 10.1002/brb3.70071 (PMC11460607; doi:10.1002/brb3.70071)

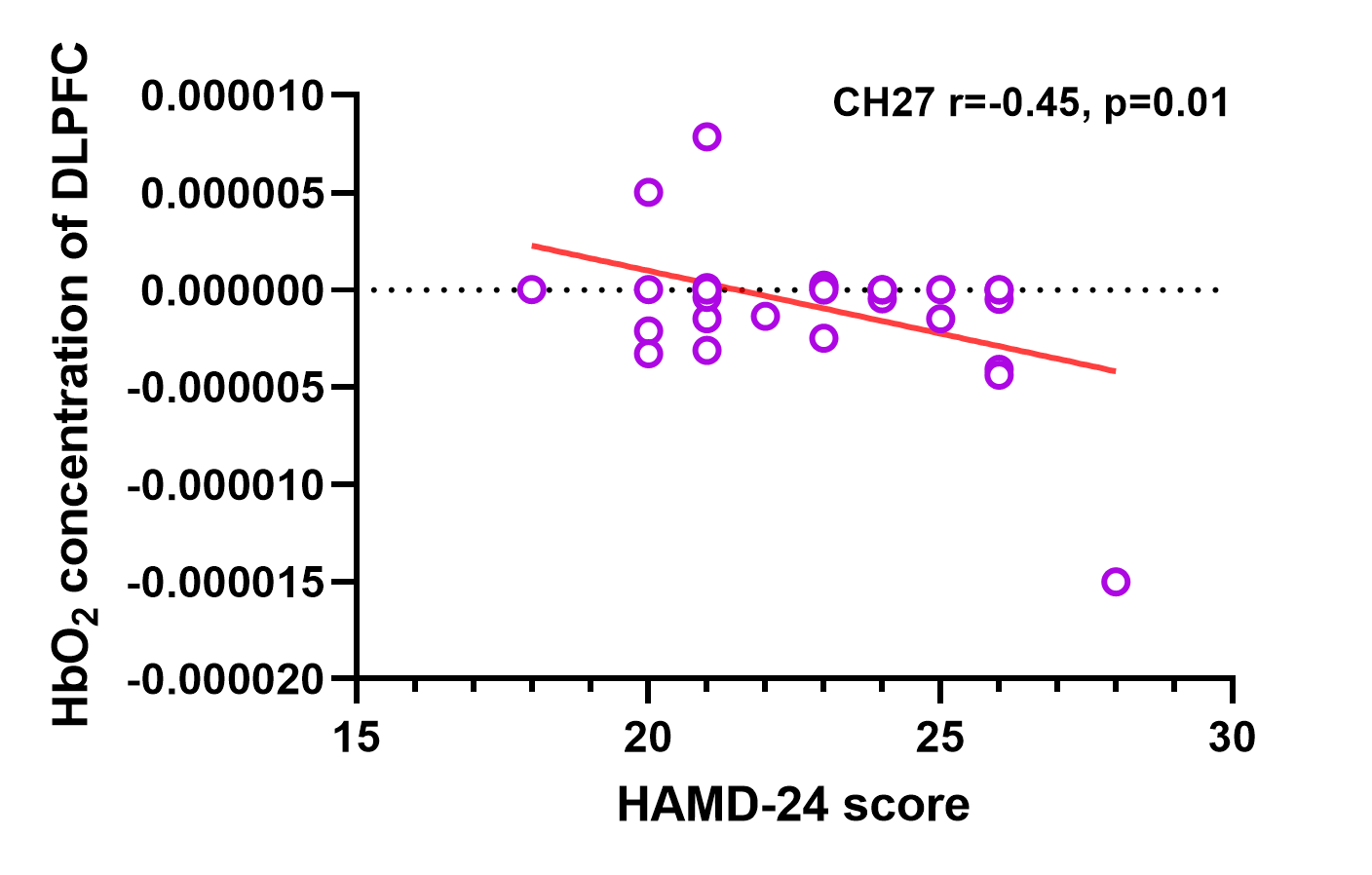

Supplement: Supplementary file 3 — Supporting Information [file BRB3-14-e70071-s004.tif]

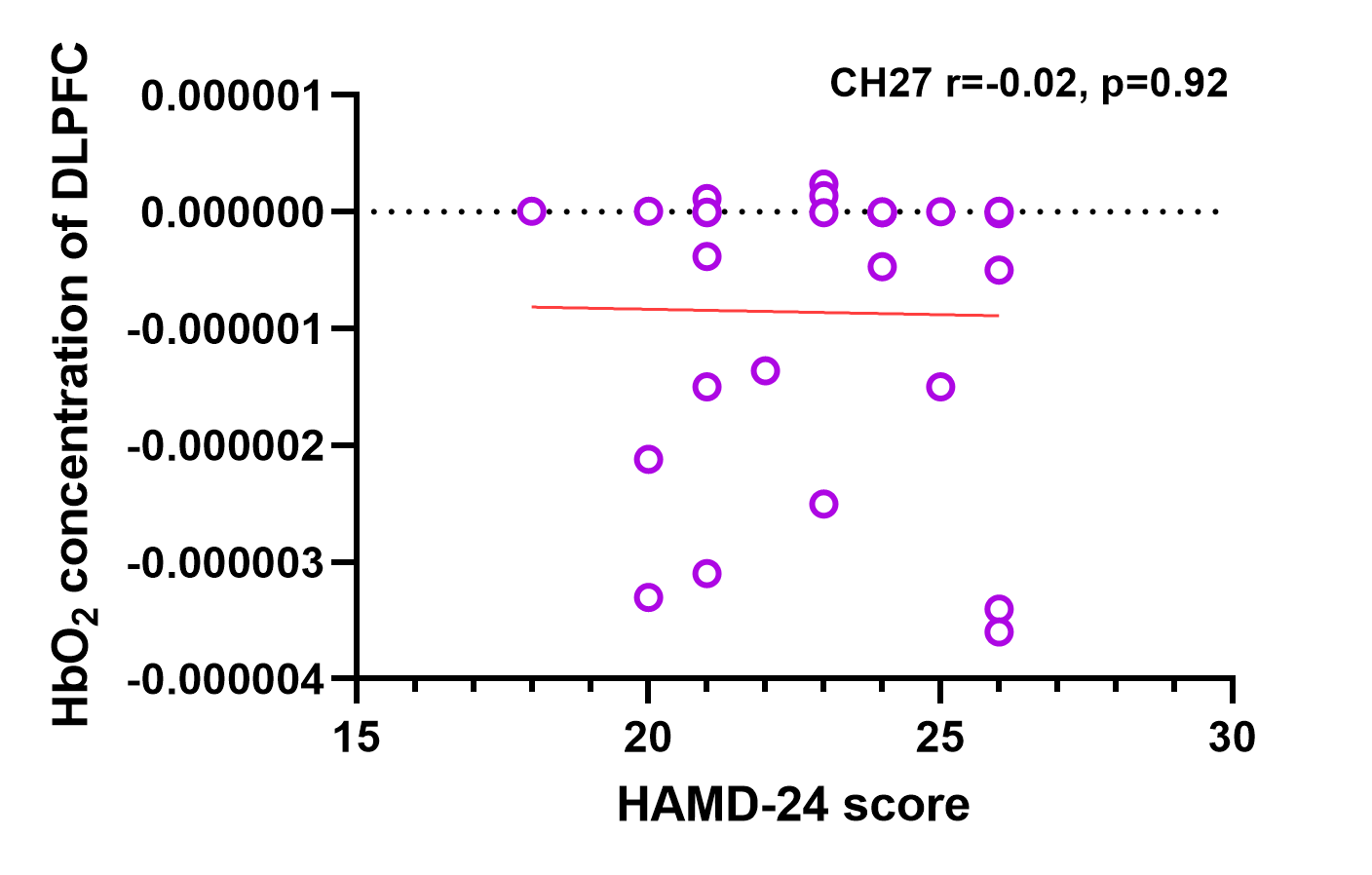

Supplement: Supplementary file 4 — Supporting Information [file BRB3-14-e70071-s001.tif]
